# Parental support and monitoring as associated with adolescent alcohol and tobacco use by gender and age

**DOI:** 10.1186/s12889-021-12119-3

**Published:** 2021-11-04

**Authors:** Rosalina Mills, Michael J. Mann, Megan L. Smith, Alfgeir L. Kristjansson

**Affiliations:** 1 School of Public Health, West Virginia University, Morgantown, USA; 2 Boise State University, Boise, USA

**Keywords:** Parental support, Parental monitoring, Adolescent substance use, Appalachia

## Abstract

**Background:**

Parental support (PS) and parental monitoring (PM) are known protective factors against adolescent substance use (SU). However, little is known about whether PS and PM may affect SU outcomes differently by gender and age. This study examined the relationship between PS and PM and adolescent SU, specifically alcohol and tobacco use, stratified by gender and age group.

**Methods:**

Middle and high school students (*n* = 2351, 48.5% Female) completed surveys of self-reported SU, perceived PS and PM, and socioeconomic background. Age group was defined dichotomously as grade 7–8 Middle school and grade 9–10 High school students. PS and PM were each measured using previously validated tools. SU was measured by lifetime and past 30 days cigarette/alcohol use. One-way ANOVA and binary logistic regression models were completed. Odds ratios and means were reported.

**Results:**

PS and PM were significantly and negatively related to all outcome variables regardless of gender and age group. Mean differences in PS and PM were insignificant between age groups. Between genders, PM scores were significantly higher for girls (14.05) compared to boys (13.48) (*p* < 0.01). Odds Ratios of all four SU types (for alcohol and tobacco use) increased with higher age group, with ORs ranging from 1.45–2.61 (*p* < .05).

**Conclusions:**

PS and PM were protective against SU for all participants, consistent with previous literature. Girls reported greater parental monitoring than boys, irrespective of age-group. While girls experienced higher levels of monitoring, they did not report lower SU than boys. This suggests that monitoring girls more closely than boys appears unnecessary in preventing adolescent SU. Finally, PS was a more significant factor in preventing SU for older adolescents (high school aged group) than for younger adolescents, irrespective of gender suggesting that PS may be more impactful and important as adolescents age. As children mature, particularly from middle school to high school, PS may play a larger role in preventing SU for older adolescents compared to younger ones.

## Introduction

Parental support and monitoring have emerged as two important tools in efforts to reduce adolescent substance use and abuse [1–5]. Although the general value of these tools has been well supported, questions remain about how these tools might apply differently to adolescents of varying ages and genders in a manner that further enhances their effectiveness. Understanding how to use parental support and monitoring in an increasingly refined and targeted manner, to prevent adolescent use of substances such as tobacco (smoking or vaping), represents an especially important opportunity to strengthen primary prevention efforts. Therefore, this study examines the associations between parental support, parental monitoring, and adolescent substance use and whether these associations differ by gender and age group among middle and high school students. Findings may provide new evidence about how to best use parental support and monitoring to prevent adolescent use of substances in order to reduce the risk of misuse, abuse or addiction in the future.

Previous research on parental support and monitoring have provided clear definitions of each concept, presented examples of effective target behaviors, and identified some general benefits of these tools when they are used to prevent adolescent substance use and abuse. A brief summary of these findings includes the following core concepts.

### Parental support

Parental support has been defined as “parental behaviors toward the child, such as praising, encouraging and giving physical affection, which indicate to the child that he or she is accepted and loved” [6]. In practice, parents with high parental support will demonstrate several qualities such as caring and warmth, willingness to provide advice, and having open discussions with their children [7]. Existing narratives show that children with low parental support often display negative emotions, [8] lack the ability to cope with stress, and more often engage in substance use [9].

Studies among adolescents have identified parental support as a protective factor for multiple outcomes such as alcohol use and other substance use, [10] depression, and anxiety [11, 12]. Parental support, such as having parents that listen, and ease of access to emotional support, is a protective factor against adolescent anxiety, [13] and is inversely related to various forms of substance use [2, 10, 14–17]. Conversely, just as high parental support is a protective factor for substance use, low parental support has been found to be associated with greater levels of substance use [12, 18].

### Parental monitoring

Similar to parental support, parental monitoring—tracking children’s whereabouts and activities—is also a known protective factor against alcohol consumption and other substance use among adolescents [1, 11, 19]. Parental Monitoring differs from Parental Support, in that monitoring pertains to parents’ knowledge of a child’s whereabouts (tracking and surveillance), while parental support pertains to emotional availability and presence [20].

Parental monitoring has been found to delay alcohol initiation in adolescents, as well as to reduce levels of later drinking [21, 22]. Lack of parental monitoring has been associated with an increased risk of engagement in alcohol use among adolescents [5, 23]. On a related note, high parental monitoring has been associated with improved health-related outcomes in adolescents; such as better mental well-being and less delinquency [24]; improved medication adherence [25]; and reduced substance use and substance use intentions among adolescents, [1, 23, 26] all of which are positively related to substance use.

### The potential benefits of stratifying by gender and age group

Previous systematic and meta-analytic research has established the effectiveness of parental monitoring and support at reducing overall adolescent substance use [27–29]. However, there are reasons to believe that parental support and monitoring may be more or less effective based on a young person’s gender or age group. While parental support and parental monitoring have been studied across multiple populations, comprehensive investigations by gender and age have been rare thus far. Studies that have examined these differences have shown inconsistent results. To date, no study has aimed to include both gender differences and age-related differences in the same model.

#### Gender

It is no secret that boys and girls are raised and socialized quite differently from each other, around the world and in the U.S. [30–32] In most familial settings, girls are generally encouraged to be more submissive, demonstrate more caution, and generally have less independence [33]. Boys, contrarily, are encouraged to be more independent, take more risks, and are often monitored less than their female counterparts [34, 35]. This difference in socialization and nurturing is evident in the levels of parental monitoring and support we see in boys and girls. Girls are generally monitored more closely by their parents and guardians than boys, and report higher parental support [36]. However, it is unclear if this additional monitoring among young women yields additional reductions in substance use.

While parental support and monitoring have generally been found to be negatively associated with adolescent substance use, stratifying the analyses by gender has produced largely inconclusive results thus far. Several studies investigating the relationship between parental support and monitoring with adolescent substance use have shown no significant differences between males and females. In those studies, greater parental support and parental monitoring were found to predict lower lifetime substance use and reduced recent substance use for both genders and no gender differences were observed [37, 38]. However, other studies have suggested that young women may benefit from higher parental support and monitoring as they reported lower levels of alcohol, cigarette and marijuana use [5, 38, 39]. Additionally, other studies have found that parental support and monitoring have larger impacts on reducing substance use among males. In middle schoolers, some evidence has shown that increased parental support is associated with decreased alcohol consumption among boys, yet found no such relationship among girls [40]. Similarly, for parental monitoring, some studies have shown that increased parental monitoring is associated with less alcohol or tobacco use among boys, but not among girls [39, 41]. Thus, findings seem incomplete regarding potential gender differences across parental monitoring and support.

#### Age group

It has been well-documented that alcohol and/or other substance use in adolescence increases the likelihood of alcohol and substance use disorders later in life, and that there are benefits associated with delaying the onset of adolescent substance use [42, 43]. However, not much is currently known about whether or not parental support and monitoring may impact younger and older adolescents differently. Very few studies have stratified by age groups in their analyses, and many well-done studies did not stratify by age even when there was an opportunity to do so [1, 44]. The few studies that have examined adolescent substance use by age group differences have been largely inconclusive. Some found no age-related differences, [45] while others found some age-related differences but did not investigate parental support or monitoring separately [46]. Likewise, studies stratifying age by grade level, examining younger and older adolescents, found that parental involvement was a protective factor against substance use for all grades, but also did not examine differences in parental involvement based on support and monitoring [47].

In sum, very few studies have examined whether parental support and monitoring differ by gender and age group in their relationship with adolescent substance use, and no study has simultaneously examined these relationships in one and the same model. Given how differently boys and girls are socialized in the US and developmental differences between younger and older adolescents, it is important to measure the differences in support and monitoring each group receives and to learn more about how these strategies may effectively prevent substance use. The current study will address this gap in the prevention research by collecting data from a large sample of middle and high school-aged girls and boys and analyzing differences in the associations between parental support and monitoring as protective factors against substance use as stratified by age group and gender.

## Methods

### Participants

This study is part of a perennial community health promotion project titled “Integrated Community Engagement (ICE) Collaborative”, designed to strengthen protective and reduce risk factors among young adolescents. Baseline data was utilized from all 16 public middle and high schools located in two West Virginia counties in the United States during fall of 2019. Students from these counties represent a spectrum of diverse characteristics from families living in severe isolation/poverty to modest privilege/affluence [48].

### Procedure

During the fall of 2019 survey data were collected by teachers under the supervision of a school contact agent that operated as a liaison to the research team. Upon review by West Virginia University Institutional Review Board (IRB), a requirement for informed consent by parents/caregivers was waived due to the benign nature of the study questions, confidentiality guarantee (no individually identifiable data was collected), and importance of very high response rates for community engagement purposes (IRB # 1406345394A009). All accessible students enrolled in grades seven through twelve were surveyed. The aggregated sample consisted of 3395 respondents from all schools in two purposively selected counties (response rate = 80.4%). One participating county is located in the Southern part of the state and the other in the central part. On the most recent Robert Wood Johnson County health rankings these counties were ranked in the bottom quarter out of the 55 counties in the state. Table 1 includes descriptive statistics for all study variables.

**Table 1:** Descriptive Statistics

*N* = 2351	Younger group, grades 7–8 (*n* = 1229)	Older group, grades 9–10 (*n* = 1122)
	n (%)	n (%)
**Gender**		
Female	596 (48.5%)	543 (48.4%)
Male	602 (49.0%)	554 (49.4%)
**Family Structure**		
Lives with both parents	630 (51.3%)	558 (49.7%)
Other living arrangements	599 (48.7%)	564 (50.3%)
**Lifetime Drunkenness (Ever been drunk)**	103 (10.0%)	222 (21.6%)
**Current Alcohol Use (Past 30 Days)**	65 (6.4%)	136 (13.4%)
**Lifetime Cigarette Use (Ever Used)**	180 (17.3%)	255 (24.5%)
**Current Cigarette Use (Past 30 Days)**	46 (4.4%)	96 (9.2%)
**Race/Ethnicity**		
Asian	17 (1.4%)	13 (1.2%)
White	1116 (90.8%)	1037 (92.4%)
Black or African American	60 (4.9%)	55 (4.9%)
Hispanic or Latino	34 (2.8%)	29 (2.6%)
Native Hawaiian or Pacific Islander	5 (0.4%)	5 (0.5%)
American Indian or Native American	76 (6.2%)	51 (4.5%)
Other	48 (4.0%)	27 (2.4%)
	Mean (SD)	Mean (SD)
**Parental Support (range 4.00–16.00)**	17.1 (3.5)	17.1 (3.7)
**Parental Monitoring (range 5.00–20.00)**	13.7 (2.5)	13.6 (2.7)
**Maternal Education (range 1.0–6.0)**	4.4 (2.8)	5.0 (2.7)
**Relative Income (range 1.0–7.0)**	5.0 (1.4)	4.9 (1.3)

### Measures

#### Dependent variables

##### Smoking status

*Lifetime smoking*

Students’ smoking history was measured with a single question “How often have you smoked cigarettes in your lifetime?”, with response categories ranging from 1 = “Never” to 7=“40 times or more”. Responses were coded into a binary variable titled with 0 = “No” and 1 = “Yes”.

*Current smoking*

Current cigarette smoking status was measured with a single question. “How much have you smoked cigarettes, on average, during the last 30 days?”. Response categories ranged from 1 = “Nothing” to 7=“More than 20 cigarettes per day”. Responses were coded into a binary variable titled with 0 = “No” and 1 = “Yes”.

*Current alcohol use*

Current alcohol use was assessed with four items that were assessed with the question “How often have you had a drink of alcohol of any kind in the past 30 days?” Response categories ranged from 1 = “Never” to 7=“40 times or more”. Responses were coded into a binary variable with 0 = “No” and 1 = “Yes”.

*Lifetime drunkenness*

Adolescent drunkenness was assessed with the question “How often have you become drunk during your lifetime”. Response categories ranged from 1 = “Never” to 7=“40 times or more”. Responses were coded into a binary variable with 0 = “No” and 1 = “Yes”.

#### Independent variables

##### Parental support

The Perceived Parental Support Scale (PPS), previously validated by Kristjánsson et al., [7] was used to assess parental support among respondents. The scale includes five items that were headed with the statement “How easy or hard would it be to receive the following from your parents/caregiver?”: [1] Caring and warmth; [2] Discussions about personal affairs; [3] Advice about school; [4] Advice about other issues (projects) of yours; [5] Assistance with other things. Response categories ranged from 1 = Very difficult to 4 = Very easy. For univariate models, scores were summed to form a scale’ total possible scores ranged from 5 to 20, with lower scores indicating lower levels of parental support. For the multivariate interaction models, scores were averaged (m = 3.39) to form a scale (α = .91).

##### Parental monitoring

Parental monitoring was assessed with four items used in previous studies [49, 50] pertaining to parental knowledge about their children’s whereabouts and their friends: [1] My parents/caregivers know whom I am with in the evenings; [2] My parents/caregivers know where I am in the evenings; [3] My parents/caregivers know my friends; [4] My parents/caregivers know the parents of my friends. Response categories ranged from 1=“Applies very well to me” to 4=“Applies very poorly to me”. For univariate models, scores were reverse coded and summed to form a scale (α = .82). Total possible scores ranged from 4 to 16, with lower scores indicating higher parental monitoring. For multivariate interaction models, scores were averaged to form a scale (m = 3.39).

##### Gender

Gender was measured with a single item: “How do you describe your gender?”, with answer options 1 = “Boy”, 2 = “Girl”, 3 = “Gender non-conforming”, and 4=“Other (Please specify)”. For the purposes of our analyses, gender was re-coded with 0 = “Boys” and 1 = “Girls”, and 85 respondents that answered “other” or “non-conforming” were omitted.

##### Age group

Age was dichotomized into two groups, signifying older and younger adolescents with grades 7–8 being classified as ‘0 = Middle School-aged’ and grades 9–10 classified as ‘1 = High School-aged’.

#### Control variables

Three variables, used in previous studies, [51, 52] were employed as proximal measures for socioeconomic status (SES). These include maternal education, family structure, and relative family income. Socioeconomic status has been associated with many adolescent health outcomes, including substance use [51–53].

##### Maternal education

Similar to some previous studies, [53] maternal education was assessed with one item: “What is the highest level of schooling your mother has completed?”, with answers ranging from 1=“I don’t know/doesn’t apply to me” to 6=“Graduated from junior college or trade school”.

##### Family structure

Family structure was reported via one multiple-choice question pertaining to all people living in the student’s household. For the purpose of our analyses we merged respondents into a dichotomized variable with 1 = “lives with both biological parents”, and 0 = “lives in other arrangements”.

##### Relative income

Relative income was measured with the question, “How well off financially do you think your family is in comparison to other families in your country?”; with answer options, reverse coded, ranging from 1=“Much worse off” to 7=“Much better off”.

### Statistical analyses

Employing one-way Analysis of Variance (ANOVA) we began our analyses by testing the mean difference on the primary independent variables of interest, parental support and parental monitoring, first by stratifying the data by age and testing within gender, and then by stratifying the data by gender and testing within age groups. These results are portrayed in the beginning of the Results section. We then proceeded to run a series of binary logistic regression models to assess the multivariate relations and potential three-way and two-way interaction effects across primary independent variables and the four outcomes; lifetime cigarette smoking, current cigarette smoking, current alcohol use, and lifetime drunkenness, while controlling for SES. Due to multicollinearity issues inherent in interaction models, we reduced multicollinearity issues by centering all variables in the final models, which kept all Variance Inflation Factors in the acceptable range (< 2). Results are portrayed in odds ratios (OR) with 99% confidence intervals (CI).

## Results

Demographic information and descriptives are included in Table 1.

We began by running a correlational analysis which showed that there were no high correlations across key study variables. All correlations were less than .2, other than substance use practices with other substance use (which were not included in the same model. Further details are in Table 2, below).

**Table 2:** Bivariate Correlations

	Gender	Relative Income	Family Structure	Lifetime Cigarettes	Current Cigarettes	Lifetime Drunkenness	Current Alcohol	Parental Monitoring	Parental Support
Gender	.	−0.06**	0.00	−0.05**	− 0.05**	− 0.03	−0.06**	0.14**	0.01
Relative Income	−0.06**	.	0.013**	−0.12**	−0.07**	0.10**	−0.04*	0.13**	0.19**
Family Structure	0.00	0.013**	.	−0.18**	−0.11**	− 0.14**	−0.07**	0.14**	0.20**
Lifetime Cigarettes	−0.05**	−0.12**	− 0.18**	.	0.54**	0.51**	0.37**	−0.20**	−0.21**
Current Cigarettes	−0.05**	−0.07**	− 0.11**	0.54**	.	0.42**	0.41**	−0.19**	−0.16**
Lifetime Drunkenness	−0.03	0.10**	−0.14**	0.51**	0.42**	.	0.61**	−0.21**	−0.17**
Current Alcohol	−0.06**	−0.04*	− 0.07**	0.37**	0.41**	0.61**	.	−0.18**	−0.12**
Parental Monitoring	0.14**	0.13**	0.14**	−0.20**	−0.19**	− 0.21**	−0.18**	.	0.31**
Parental Support	0.01	0.19**	0.20**	−0.21**	−0.16**	− 0.17**	−0.12**	0.31**	.

*P < .05

**P < .01

Next, we tested for mean differences across parental support and parental monitoring by gender within the two age groups. For the younger group, 7-8th grade students, the mean score on parental support was 17.19 (SD = 3.45) for boys and 17.23 (SD = 3.32) for girls with a non-significant F-test for group differences [*F*(1,1134) = 0.40, *p* = .84]. Parental monitoring scores within the same age group were 13.48 (SD = 2.56) for boys and 14.05 (SD = 2.24) for girls which is significant at the 95% level [*F*(1,1147) = 16.08, *p* < .01].

For the older group, 9-10th grade students, the mean score on parental support was 17.23 (SD = 3.40) for boys and 17.01 (SD = 3.84) for girls, also with a non-significant F-test for group differences [*F*(1,1074) = 1.09, *p* = .30]. Parental monitoring scores within the older age group were 13.31 (SD = 2.75) for boys and 13.83 (SD = 2.63) for girls which is significant at the 95% level [*F*(1,1063) = 9.91, *p* = .02]. Subsequent tests for mean differences by age group within gender revealed non-significant differences for boys on parental support [*F*(1,1111) = 0,06, *p* = .82] and parental monitoring [*F*(1,1111) = 1.13, *p* = .29] as well as for girls on parental support [*F*(1,097) = 1.03, *p* = .31] and parental monitoring [*F*(1,1099) = 2.24, *p* = .14]. Table 1 includes descriptive statistics for all study variables.

Frequency (percentage) or Mean (SD) reported.

Table 3 includes the results from the logistic regression models to assess the relations between the primary independent variables, parental monitoring and parental support, and all four outcome variables.

**Table 3:** Binary Logistic Regression Models (Odds Ratios with 95% Confidence Intervals)

	Outcomes Variable for each Model			
	Lifetime Cigarettes	Current Cigarettes	Current Alcohol	Lifetime Drunkenness
Age Group [0 = younger, 1 = older]	1.45* [1.14, 1.84]	2.25* [1.49, 3.41]	2.20* [1.56, 3.10]	2.61* [1.98, 3.46]
Gender [0 = boy, 1 = girl]	1.00 [0.79, 1.27]	1.05 [0.72, 1.54]	0.68* [0.49, 0.94]	0.93 [0.71, 1.20]
Family Structure [0 = other, 1 = both parents]	0.57* [0.45, 0.73]	0.79 [0.53, 1.18]	0.80 [0.57, 1.12]	0.73* [0.56, 0.96]
Maternal Education	0.99 [0.95, 1.04]	0.95 [0.88, 1.02]	1.10* [1.04, 1.17]	0.99 [0.94, 1.04]
Relative Income	0.88* [0.80, 0.96]	0.94 [0.81, 1.08]	1.02 [0.90, 1.15]	0.89* [0.80, 0.98]
Parental Support	0.91* [0.88, 0.94]	0.91* [0.87, 0.96]	0.92* [0.88, 0.96]	0.92* [0.89, 0.95]
Parental Monitoring	0.88* [0.85, 0.93]	0.86* [0.81, 0.92]	0.87* [0.82, 0.91]	0.89* [0.85, 0.93]

*p < .05

As shown, parental support and parental monitoring were significantly and negatively related to all outcome variables with ORs ranging from .91–.92 for parental support (*p* < .05) and ORs ranging from .86–.89 for parental monitoring (*p* < .05). Further, the odds of all four types of substance use, lifetime cigarette smoking, current cigarette smoking, current alcohol use, and lifetime drunkenness, increase with higher age group with ORs ranging from 1.45–2.61 (*p* < .05).

Finally, we ran the four models testing for three-way interaction effects of Monitoring or Support with Gender and Grade as well as two-way interaction effects (see Table 4). There were no significant three-way effects. Only two interaction effects were significant. Significant interaction relations were found for Parental Support and Age for current cigarette use (OR = 1.94, 99% CI = 1.04–3.63) and current alcohol use (OR = 1.86, 99% CI = 1.07–3.22). The main effect of parental monitoring was significantly associated with all substance use outcome variables. The main effect of parental support was significantly associated with both lifetime substance use outcomes.

**Table 4:** Binary Logistic Regression Models (Odds Ratios with 99% Confidence Intervals) with Interaction Terms

	Outcomes Variable for each Model			
	Lifetime Cigarettes	Current Cigarettes	Current Alcohol	Lifetime Drunkenness
Age Group [0 = younger, 1 = older]	2.52* [1.12, 2.10]	2.63* [1.45, 4.78]	2.60* [1.59, 4.26]	2.73* [1.87, 3.98]
Gender [0 = boy, 1 = girl]	0.97 [0.71, 1.33]	1.05 [0.61, 1.79]	0.71 [0.46, 1.11]	0.91 [0.64, 1.28]
Family Structure [0 = other, 1 = both parents]	0.56* [0.41, 0.76]	0.72 [0.43, 1.20]	0.81 [0.52, 1.24]	0.72* [0.50, 1.02]
Maternal Education	0.99 [0.94, 1.05]	0.95 [0.87, 1.05]	1.11* [1.02, 1.20]	1.00 [0.93, 1.06]
Relative Income	0.87* [0.78, 0.98]	0.93 [0.77, 1.12]	1.00 [0.85, 1.17]	0.88* [0.77, 1.00]
Parental Support	0.64* [0.52, 0.79]	0.58* [0.42, 0.81]	0.60* [0.45, 0.80]	0.64* [0.50, 0.82]
Parental Monitoring	0.61* [0.48, 0.77]	0.57* [0.39, 0.81]	0.54* [0.40, 0.75]	0.62* [0.47, 0.81]
Parental Support* Age Group	1.44 [0.96, 2.18]	1.94* [1.04, 3.63]	1.86* [1.07, 3.22]	1.36 [0.85, 2.17]
Parental Support* Gender	0.77 [0.51, 1.17]	0.96 [0.52, 1.75]	0.90 [0.52, 1.54]	0.83 [0.52, 1.33]
Parental Monitoring* Age Group	0.96 [0.60, 1.53]	0.83 [0.41, 1.69]	1.00 [0.54, 1.86]	0.99 [0.58, 1.69]
Parental Monitoring* Gender	1.07 [0.66, 1.71]	0.76 [0.37, 1.56]	0.96 [0.51, 1.79]	0.99 [0.58, 1.70]

*P < .01

## Discussion

This investigation into gender and age-related differences in parental support and parental monitoring on alcohol and tobacco use revealed four important findings. First, at the bivariate level, girls reported more parental monitoring than boys irrespective of age-group, while no differences were observed for parental support by gender. This coincides with existing research given the differing socialization of boys and girls; girls are more closely monitored than boys. Further, no differences were observed for either parental support or parental monitoring by age-group within gender. In short, younger boys are not supported more or monitored more by their parents than older boys, and the same goes for girls.

Second, the initial multivariate analyses revealed both parental support and parental monitoring were found to be protective of substance use irrespective of age group and gender. That is, for both boys and girls in both the younger and older groups, greater levels of parental support and parental monitoring served to decrease the odds of cigarette smoking and alcohol use across all four alcohol and tobacco categories.

Third, we observed no gender differences between parental support and parental monitoring on all four outcome variables. In other words, the associations between parental support and parental monitoring on the four substance use outcomes were not different for boys or girls irrespective of age group.

Finally, we found a significant difference in the associations between parental support and three of four outcome variables by age group, with stronger associations for the older group. Put simply, increased parental support appears to be stronger protection for lifetime cigarette smoking, current cigarette smoking, and current alcohol use among older adolescents (9-10th graders) than when compared with the younger adolescents (7-8th graders) after taking account of parental monitoring and control variables. No such differences were observed for parental monitoring.

### Implications for Research and Practice

 While girls are monitored at higher levels than boys, regardless of age; parental monitoring does not appear to affect one gender over the other in terms of substance use outcomes. It is common for girls in the US and Europe to be monitored more closely than their male counterparts [54, 55]. However, this study shows that the extra monitoring experienced by girls in this population does not necessarily indicate a stronger protective factor against substance use. While girls are often monitored more closely than boys, it does not necessarily result in a stronger protective factor against substance use. These findings suggest monitoring girls more closely than boys appears unnecessary in terms of preventing adolescent substance use and abuse. Taken together, our findings indicate the importance of parental monitoring throughout adolescent development, regardless of a child’s age or gender. It appears that monitoring can have a significant impact on adolescent onset of substance use. Thus, regardless of the expectation of substance use across age or gender, parents who are aware of where their children are and who they are with can have an impact on substance use initiation.

While gender differences were not observed in this study, a significant interaction effect was found for support by age group in three of four outcomes, favoring older participants. It has been well established that parental monitoring and parental support are protective factors against substance use outcomes among teens and adolescents, [1–3] and our study arrived at those conclusions as well. However, this study also found that parental support is an even stronger protective factor against substance use for older adolescents than it is for younger adolescents. This implies that as children mature, particularly from middle school to high school, parental emotional support may play an even bigger role in preventing substance use for the older youth compared to the younger ones. As further demonstrated in our models, with age comes a natural progression in the odds of substance use. This is to be expected given that opportunities to experiment with tobacco and alcohol likely increase with age and increased independence. Our findings demonstrate that the protective effect of parental support on adolescent experimentation with tobacco and alcohol is even greater as they move into high school, a period associated with more independence and opportunities to participate in risk behavior [56]. While parental monitoring has long been regarded as an important protective factor for adolescent substance use, [1, 10] our observation indicates that parental support may grow in importance in preventing substance use among this population. The importance of parental support as children age may be an important aspect to investigate in future studies.

### Strengths and limitations

This study had some strengths. First, we employed a large sample which allowed us to confidently stratify results by age group and gender while retaining suitable statistical power. Second, the wide age range in the sample also allowed us to capture age differences and separate statistical tests for older and younger adolescents, and to do so separately for boys and girls. Our study also had several limitations. The sample population consisted exclusively of public school youth in two West Virginia counties, and may not be generalizable to the larger American population. Secondly, the cross-sectional nature of our data limits our ability to draw conclusions concerning cause and effect. Third and finally, all responses were self-reported and thus subject to recall bias.

#### Conclusions and future directions

This study reinforces the value of parental support and monitoring as effective strategies that help prevent and reduce adolescent substance use and abuse. Additionally, findings emphasize that, although a baseline level of consistent parental monitoring remains important throughout adolescence, increasing levels of parental support as young people grow older and become more independent seems to be protective for both genders. As such, ensuring parents understand how to provide effective support represents an essential component of primary prevention-based efforts to reduce adolescent substance use and abuse. Findings also suggest that – at least in terms of adolescent alcohol and tobacco use – parents may unnecessarily over-monitor young women’s activities. However, it is difficult to definitively make this determination without additional research that accounts for all substances, and for the different types of parental monitoring and support offered to different genders.

Although this study provides important evidence related to the overall value of parental support and monitoring at different ages and by gender, it did not investigate the relative value of specific monitoring and support behaviors (i.e., emotional comfort vs. advice). Future studies designed to extend this line of inquiry could include the influence and relative value of specific parental support and monitoring strategies, especially those related to providing parental support at different ages. Doing so may represent an important next step in better understanding how to apply these primary prevention strategies in the most effective manner possible.

## Data Availability

The datasets generated and/or analyzed during the current study are not publicly available due co-utility and ownership by local schools and boards of education but are available from the corresponding author on reasonable request without school, county, or individual identifiers.
